# The Unhappy Mental Health Triad: Comorbid Severe Mental Illnesses, Personality Disorders, and Substance Use Disorders in Prison Populations

**DOI:** 10.3389/fpsyt.2020.00804

**Published:** 2020-08-14

**Authors:** Adrian P. Mundt, Gergő Baranyi

**Affiliations:** ^1^Faculty of Medicine, Universidad Diego Portales, Santiago, Chile; ^2^Department of Psychiatry, Faculty of Medicine, Hospital Clínico Universidad de Chile, Santiago, Chile; ^3^Centre for Research on Environment, Society and Health, School of GeoSciences, The University of Edinburgh, Edinburgh, United Kingdom

**Keywords:** prison population, prevalence, severe mental illness (SMI), comorbid addictions, personality disorder (PD), substance use disorder (SUD)

## Abstract

**Background:**

Information on the comorbidity of mental health problems in prison populations is scarce. The aim of the present study was to assess the prevalence of comorbidities at intake to prison between three diagnostic groups: severe mental illnesses (SMIs), personality disorders (PDs), and substance use disorders (SUDs). The co-occurrence of those disorders in prison populations may require the integration of differential treatment approaches and novel treatment trials.

**Methods:**

A consecutive sample of N = 427 (229 male and 198 female) individuals committed to imprisonment in Santiago de Chile was assessed with the Mini Neuropsychiatric Interview and the Structured Clinical Interview for DSM-IV (module for borderline personality disorders) on arrival at prison. Diagnoses were *a priori* grouped as SMI including psychosis, bipolar disorder and major depression, PD including borderline and antisocial PD and SUD including alcohol and drug abuse or dependence. Sex stratified multivariate binary logistic regression analyses were conducted to assess sociodemographic, criminal and treatment characteristics of individuals with at least one diagnosis from each of the three diagnostic groups.

**Results:**

The triad of SMI, PD, and SUD was present in n = 138 (32.3%; 95% IC: 28.0–36.9) study participants, n = 105 (45.9%; 95% CI: 39.4–52.4) of the men and n = 33 (16.7%; 95% CI: 12.1–22.6) of the women. Among those with the disorder triad, n = 129 (30.2%; 95% CI: 26.0–34.8) had major depression, PD and SUD; n = 54 (12.6%; 95% CI: 9.8–16.2) had psychosis, PD and SUD. The disorder triad was more common in men (OR = 4.86; 95% IC: 2.63–8.95), younger age (OR = 0.94; 95% CI: 0.91–0.97), and participants with lower educational levels (OR = 1.69; 95% CI: 1.01–2.82). The disorder triad was significantly associated with previous incarcerations (OR 2.60; 95% CI: 1.55–4.34) and histories of psychiatric hospitalizations (OR 2.82; 95% CI: 1.27–6.28).

**Discussion:**

The complex triad of disorders from different diagnostic groups is common in prison populations, especially among young men. Successful treatment interventions may have the potential to break a cycle of repeat institutionalization in prisons and psychiatric institutions.

## Introduction

Over 11 million people are held in prisons throughout the world at any given time (1). A range of studies including meta-analyses established prevalence estimates for single psychiatric disorders that were consistently higher than in the general populations (2). The prevalence of psychosis was estimated to be 3.6% in male and 3.9% in female prisoners, while the prevalence of major depression was 10.2 and 14.1% respectively (3). High rates of alcohol (24%) and drug use disorders (30–51%) were present in prison populations on intake to the facility (4, 5). Personality disorders (PDs) were reported to be common in prison populations, with borderline personality disorders present in approximately half of the population (6, 7). Antisocial personality disorder was present in more than half of the young male prison population and in 90% of those with childhood onset conduct disorder (8).

However, in order to better understand the treatment and care needs, it is necessary to assess the co-occurrence of several mental health and substance use problems in prison populations. Comorbidity has been reported in different ways for differential purposes: First, it has been reported as the number of disorders co-occurring and covered by the given instrument used in a study (9–11), which serves the purpose of identifying groups with multiple disorders, polymorph psychopathology, and unfavorable outcomes. However, assessing the quantity of diagnoses has the disadvantage that it does not distinguish between the types of comorbidity. For example, two comorbid anxiety disorders may be less complex to treat and less relevant than comorbid major depression with psychotic symptoms and illicit drug dependence. A second way to approach comorbidity among prison populations is to *a priori* group disorders based on similarity in treatment needs or psychopathology and assess co-occurrence across those groups, *i.e.* severe mental illness (SMI) and substance use disorders (SUDs) (12) or SUD and other disorders (13) or SUD, internalizing disorders and behavioral disorders (14). This approach generates prevalence estimates for specific combinations of disorders that are potentially relevant for clinical management and are comparable between settings. A third approach to report comorbidity is statistically driven, generating groups derived from the data, such as latent class analysis of diagnoses (15). This allows further understanding of the complexity of psychopathology and how the diagnoses relate to each other. Four groups were derived from latent class analyses of diagnoses in prison populations, including no comorbidities, internalizing disorders, externalizing disorders, and one group with multiple co-occurring disorders representing 11% of the prisoner population, which may pose particular clinical challenges and face scarcity of evidence for treatment interventions (15).

In all, the most pragmatic and clinically useful approach to address comorbidity may be to *a priori* aggregate single diagnoses in diagnostic groups that require similar treatment (14, 16): SMIs including major depression and psychosis regularly require psychiatric evaluation and medication. PDs often require psychotherapies such as dialectic behavioral therapy and SUD motivational interviewing, relapse prevention, and for some people, temporarily controlling the access to substances. Especially, the co-occurrence of disorders across those diagnostic groups may pose additional challenges for treatment planning and integration (17) and for prognosis, whereas comorbid disorders within specific diagnostic groups can often be treated using one single approach and thus are less relevant for treatment planning. Focusing among the axis one disorders on the severe disorders and treating them as a group is pragmatic and in line with international research on interventions for co-occurring mental health and substance use disorders in general (18, 19) and in prison populations (16). Individuals with antisocial PD may differ from those with borderline PD regarding the criminal profiles: whereas people with antisocial PD tend to show more instrumental violence, people with borderline PD commit more often emotional violence as a result from impulsivity (20). Nevertheless, grouping several personality disorders for the assessment of clinically relevant comorbidity as separate from substance use disorders is common in research and practice (21). The aim of the present study was to assess the prevalence of co-occurring disorders across the three *a priori* defined diagnostic groups SMI, PD, and SUD in prison populations. For those with the complex triad of disorders from each of the diagnostic groups, we aimed to assess socio-demographic and criminal characteristics.

## Methods

### Sample

Study participants were recruited from consecutively admitted prison populations in the three remand prison facilities serving the metropolitan region of Santiago de Chile: Santiago Uno, which serves as a central facility for admissions of male prisoners and Centro Penitenciario Feminino (CPF) San Joaquín and CPF San Miguel serving female prisoners. The interviews were usually conducted within the first week of imprisonment (median 5 days; mean 7.7 days after imprisonment). There were 470 potentially eligible individuals for the study. Seven were excluded due to mental or psychological incapacities to participate. Thirty rejected participation; six prematurely ended the interview and were excluded from analysis; data of 427 participants were used in the analyses. The rejection rate was 7.0%.

### Instruments

Age and sex were assessed using structured questions. Relationship status indicated whether respondents were living alone or with somebody else. The highest educational level was first classified according to the International Standard Classification of Education (ISCED) (22) and then divided into primary or lower (ISCED levels 0 and 1) *versus* secondary or higher (ISCED levels 2–6) educational levels. Employment status had two categories indicating working for income and not working for income (unemployed, house keeping). The type of criminal offense was classified into categories: property offense, violent crime, drug use or trafficking, possession of firearm, sexual offense, or others. The number of previous psychiatric hospitalizations and previous incarcerations was recorded and coded as present or absent. All information was derived from the interviews. We did not have access to the health or criminal files of the study participants.

The Mini-International Neuropsychiatric Interview (MINI) was used in the Spanish version to assess mental health and substance use disorders (23) to classify axis I mental disorders and antisocial personality disorder according to the fourth version of the Diagnostic and Statistical Manual of Mental Disorders (DSM-IV). We further used the module for borderline personality disorder of the Structured Clinical Interview for DSM-IV (SCID-II).

### Procedure

All female intakes to the prison system of the metropolitan region in Santiago, Chile and every third man on the daily intake lists were approached for study participation. Potential participants were first approached by the prison guard on call assisting in the logistics of the study and when available brought to the interview area of the institution for information on the study. We aimed to recruit 200 participants of each gender. Interviews were conducted by four clinical psychologists and a trained nurse and supervised by a senior consultant psychiatrist in using the instruments. In order to improve inter-rater concordance, pairs of interviewers conducted the first 20 interviews together. The interviewers alternated the lead of the interview and discussed points of discordance. The interviews lasted for 45–60 min and were held in a separate room of the prison to ensure confidentiality. The data collection took place between February 2013 and September 2013. There was a complete discussion of the study with potential participants, and all participants provided written informed consent after this discussion. The authors assert that all procedures contributing to this work comply with the ethical standards of the relevant national and institutional committees on human experimentation and with the Helsinki Declaration of 1975, as revised in 2008. The study was approved by the Ethics Review Board of the University of Chile (Acta de Aprobación 01 from 25.01.2012) and by the Ministry of Justice of the Republic of Chile (reference: Subsecretaria de Justicia 15.03.2012). For further details on the study procedures see (7).

### Analyses

We estimated prevalence rates with 95% Confidence Intervals (95% CI) for the diagnostic groups severe mental illnesses, personality disorders, substance use disorders, and for different types of comorbidities. Current major depression, bipolar disorders and/or psychotic disorders were aggregated as the diagnostic group of SMI. The decision to manage SMI as one group for the assessment of comorbidity was not based on homogeneity in the psychopathology, but the consideration that either psychosis or major depression co-occurring with SUD and/or PD can complicate clinical management and prognosis. Borderline personality disorder and/or antisocial personality disorder was aggregated as the diagnostic group of PD. Drug and/or alcohol abuse and dependence were aggregated in the diagnostic group SUD. The grouping of disorders and juxtaposition with others was *a priori* and treatment oriented, not based on statistical measures and not derived from the data.

We assessed the prevalence of dual disorders with at least one disorder from two diagnostic groups, irrespectively of whether a disorder from the third group was present or not. We also assessed the prevalence of having at least one disorder from each of the three groups. For the co-occurring disorder triad of SMI, PD, and SUD, we further explored the risk profile, separately for male and female prisoners. First, sociodemographic and criminal characteristics as well as indicators of previous institutionalization were described among prisoners with and without the comorbid triad, using chi-square tests and t-tests to test for significance. Secondly, a multivariate logistic regression model was used to examine which covariates predicted the presence of the comorbid disorder triad. Strong correlation between explanatory variables may cause biased regression coefficients; therefore, we tested for multicollinearity with the variance inflation factor (<10) and tolerance scores (>0.1). As the criminal offense of ‘drug use and trafficking’ showed high multicollinearity in the female subsample, we dropped this variable from the model run on the female subsample.

All statistical analyses were performed with Stata, version 13.

## Results

The triad of SMI, PD, and SUD was present in n = 138 (32.3%) of the total prison population, in n = 105 (45.9%) of the male, and n = 33 (16.7%) of the female study participants. The larger part of those individuals n = 129 (30.2%) had MD, PD, and SUD; the smaller part n = 54 (12.6%) had psychosis, PD, and SUD. Dual disorders from any two of the three diagnostic groups (irrespective of the presence of a disorder from the third group) were present in about 40%, and a single disorder from any of the three categories was present in more than half of the prison population. Dual disorders of severe mental illness co-occurring with PD and SMI co-occurring with SUD were present in n = 178 (41.7%) and n = 168 (39.2%) respectively. Co-occurring personality disorder and substance use disorder were present in n = 179 (41.9%; **Table 1**). Prevalence rates of dual disorders and disorder triads are also shown separately for male and female prisoners. Those rates were in general higher in men than in women.

**Table 1:** Comorbidities between different groups of disorders: 1. Severe mental disorders (SMI) including psychosis, major depression (MD) and bipolar disorders; 2. Personality disorders (PD) including borderline personality disorders (BPD) and antisocial personality disorders (APD); 3. Substance use disorders (SUD), including alcohol and drug use disorders.

Groups of disorders	Total(n = 427)			Male(n = 229)			Female(n = 198)		
	n	%	95% CI	n	%	95% CI	n	%	95% CI
SMI	251	58.8	54.0–63.4	143	62.4	55.9-68.5	108	54.4	47.5-61.4
Psychosis	68	15.9	12.7-19.7	51	22.3	17.3-28.2	17	8.6	5.4-13.4
MD	233	54.6	49.8-59.3	137	59.8	53.3-66.0	96	48.5	41.5-55.5
PD	231	54.1	49.3-58.8	163	71.2	64.9-76.7	68	34.3	28.0-41.3
BPD	216	50.6	45.7–55.3	154	67.2	61.1–73.8	62	31.3	24.8–38.4
APD	126	29.5	25.3–34.0	91	41.9	35.4–48.0	30	15.2	10.1–20.2
SUD	237	55.5	50.7-60.2	173	75.6	69.5-80.7	64	32.3	26.1-39.2
**Comorbidities between groups of disorders**									
SMI and PD	178	41.7	37.1–46.4	124	54.1	47.6–60.5	54	27.3	21.5–34.0
Psychosis and PD	62	14.5	11.5–18.2	49	21.4	16.5–27.2	13	6.6	3.8–11.0
MD and PD	167	39.1	34.6–43.8	119	52.0	45.4–58.4	48	24.2	18.7–30.8
SMI and SUD	168	39.3	34.8–44.1	120	52.4	45.9–58.8	48	24.2	18.7–30.8
Psychosis and SUD	59	13.8	10.8–17.4	46	20.1	15.4–25.8	13	6.6	3.8–11.0
MD and SUD	153	35.8	31.4–40.5	114	49.8	43.3–56.3	39	19.7	14.7–25.9
PD and SUD	179	41.9	37.3–46.7	138	60.3	53.7–66.4	41	20.7	15.6–27.0
BPD and SUD	170	39.8	35.3–44.6	130	56.8	50.2–63.1	40	20.2	15.1–26.4
APD and SUD	111	26.0	22.0–30.4	91	39.7	33.6–46.2	20	10.1	6.6–15.2
SMI, PD and SUD	138	32.3	28.0–36.9	105	45.9	39.4–52.4	33	16.7	12.1–22.6
Psychosis, PD and SUD	54	12.6	9.8–16.2	44	19.2	14.6–24.9	10	5.1	2.7–9.2
Psychosis, BPD and SUD	54	12.6	9.8–16.2	44	19.2	14.6–24.9	10	5.1	2.7–9.2
Psychosis, APD and SUD	38	8.9	6.5–12.0	33	14.4	10.4–19.6	5	2.5	1.0–6.0
MD, PD and SUD	129	30.2	26.0–34.8	100	43.7	37.3–50.2	29	14.6	10.3–20.3
MD, BPD and SUD	125	29.3	25.1–33.8	97	42.4	36.1–48.9	28	14.1	9.9–19.8
MD, APD and SUD	86	20.1	16.6–24.2	72	31.4	25.7–37.8	14	7.1	4.2–11.6

First, prevalence rates of dual disorders are reported considering three different combinations out of the three disorder groups (SMI and PD; SMI and SUD; PD and SUD). Dual disorders are considered prevalent irrespective of whether any disorder from the third group is present or not. Secondly, those with the disorder triad with at least one disorder from each of the three groups are reported. Under the prevalence of dual and triple disorders from different groups, the combination for specific (mutually non-exclusive) diagnoses with any disorder from a different group is reported using line indents.

**Table 2** shows descriptive statistics and bivariate analyses for prison populations with the triad of SMI, PD, and SUD compared to those who do not present the disorder triad. Results are shown for the total population and separately for male and female prisoners. Prison population with the disorder triad were younger, less likely to be living with a partner, more likely to be charged for any violent crime, and less likely to be charged for any drug associated crime. Furthermore, individuals with the disorder triad had more likely been previously imprisoned and received psychiatric hospital treatment. The differences in age, previous imprisonment, and psychiatric hospitalization were observed in both male and female subsamples. The differences in educational level, charges for violent crime, and drug associated crime were only seen in the female population, whereas men with the disorder triad were more likely to be unemployed prior to imprisonment (**Table 2**).

**Table 2:** Sociodemographic and criminal characteristics of male and female prisoners with and without the comorbid triad of mental disorders: severe mental illnesses (SMIs), personality disorders (PDs) and substance use disorder (SUD).

Variable	Total					Male					Female				
	Without triad(n = 289)		With triad(n = 138)		*p*	Without triad(n = 124)		With triad(n = 105)		*p*	Without triad(n = 165)		With triad(n = 33)		*p*
	n	%	n	%		n	%	n	%		n	%	n	%	
Age, mean (SD)^a^	34.0	(12.1)	26.6	(7.9)	***	33.0	(13.5)	26.4	(7.83)	***	34.7	(11.1)	27.2	(8.2)	***
Educational level															
Primary or lower	133	46.0	71	51.5		42	33.9	44	41.9		91	55.1	27	81.8	
Secondary or higher	156	54.0	67	48.5		82	66.1	61	58.1		74	44.9	6	18.2	**
Employment status															
Yes	230	79.6	101	73.2		112	90.3	83	79.0		118	71.5	18	54.6	
No	59	20.4	37	26.8		12	9.7	22	21.0	*	47	28.5	15	45.5	
Relationship status															
Living alone	147	50.9	85	61.6		61	49.2	65	61.9		86	52.1	20	60.6	
Living with partner	142	49.1	53	38.4	*	63	50.8	40	38.1		79	47.9	13	39.4	
Offense category^b^															
Property	66	22.8	40	29.0		36	29.0	29	27.6		30	18.2	11	33.3	
Violence	68	23.5	59	42.8	***	46	37.1	48	45.7		22	13.3	11	33.3	**
Drug	128	44.3	22	15.9	***	18	14.5	13	12.4		110	66.7	9	27.3	***
Firearm	5	1.7	4	2.9		3	2.4	4	3.8		2	1.21	0	0.0	
Sexual	9	3.1	5	3.6		9	7.3	5	4.8		0	0.0	0	0.0	
Previous imprisonment															
No	162	56.1	60	43.5		81	65.3	52	49.5		81	49.1	8	24.2	
Yes	127	43.9	78	56.5	*	43	34.7	53	50.5	*	84	50.9	25	75.8	**
Previous psychiatric hospitalization															
No	272	94.1	118	85.5		119	96.0	91	86.7		153	92.7	27	81.8	
Yes	17	5.9	20	14.5	**	5	4.0	14	13.3	*	12	7.3	6	19.2	*

*p < 0.05; **p < 0.01; ***p < 0.001.

^a^Variable encounters 10 missing cases.

^b^Categories are not mutually exclusive.

In the regression model testing, the link with sociodemographic variables for the whole sample, male sex (OR = 4.86; 95% IC: 2.63–8.95; p < 0.001), young age (OR = 0.94; 95% CI: 0.91–0.97; p < 0.001), and lower educational level (OR = 1.69; 95% CI: 1.01–2.82; p < 0.05) was identified as risk factors for the disorder triad (**Table 3**). Furthermore, the disorder triad was associated with previous imprisonment (OR 2.60; 95% CI: 1.55–4.34; p < 0.001) and previous psychiatric hospitalization (OR 2.82; 95% CI: 1.27–6.28; p < 0.001) indicating an increased risk of repeat institutionalization in this population (**Table 3**).

**Table 3:** Multivariate associations of sociodemographic, criminal and psychiatric treatment variables with the triad of severe mental illness, personality and substance use disorder in prison population presented as odds ratios with 95% confidence intervals.

Variables	Total (n = 417)		Male (n = 219)		Female (n = 198)	
	OR	95% CI	OR	95% CI	OR	95% CI
Sex						
Male	4.86	2.63–8.95***	N/A		N/A	
Female	ref					
Age	0.94	0.91–0.97***	0.94	0.91–0.97***	0.92	0.87–0.98**
Education						
Primary or lower	1.69	1.01–2.82*	1.23	0.65–2.32	3.14	1.15–8.56*
Secondary or higher	ref		ref		ref	
Employment status						
Yes	ref		ref		ref	
No	1.63	0.89–2.99	1.91	0.81–4.50	1.27	0.50–3.17
Relationship status						
Living alone	ref		ref		ref	
Living with partner	0.78	0.47–1.28	0.78	0.42–1.45	0.71	0.28–1.79
Offence category						
Property	0.83	0.29-2.34	0.94	0.28–3.19	1.41	0.49–4.03
Violence	1.15	0.41–3.22	1.11	0.33–3.67	3.77	1.24–11.50*
Drug	0.63	0.21-1.88	1.40	0.35–5.26	^a^	
Firearm	1.42	0.26–7.94	2.10	0.32–14.01	^b^	
Sexual	1.17	0.24–5.73	1.48	0.28–7.86	^b^	
Previous imprisonment						
No	ref		ref		ref	
Yes	2.60	1.55–4.34***	2.50	1.34–4.68**	4.35	1.58–12.00**
Previous psychiatric hospitalization						
No	ref		ref		ref	
Yes	2.82	1.27–6.28***	4.65	1.40–15.38*	1.34	0.38–4.73

*p < 0.05; **p < 0.01; ***p < 0.001.

^a^Dropped because of multicollinearity.

^b^Dropped because no case was present.

## Discussion

About one third of our sample presented a disorder triad of severe mental disorder, personality disorder, and addiction at the moment of intake to prison in Santiago, Chile. This triad was more common among young men with lower educational levels and it was associated with indicators of previous institutionalization.

In concordance with previous findings, our results indicate that comorbid mental health and substance use disorders are common among prisoners. High rates of comorbidity have been shown for borderline personality disorder with both major depression (82%) and with substance dependence (73%) in prison populations (24). Prisoners with SMI also had high rates of SUD (12, 13, 25). For female prison populations with substance dependence, the prevalence of major depression was reported to be 62% in Mexico (26). About half of the prisoner populations with substance use disorders were reported to also have depression and/or anxiety disorders (13, 27). And *vice versa*, rates of substance use disorders were reported to be above 75% among prisoner populations with depression and anxiety disorders (28, 29). In contrast to our study, others evaluated the prevalence data over the lifespan. The lifetime prevalence of substance use disorders among prisoners with lifetime prevalence of psychosis may be up to 90% (30). Co-occurring mental disorders and substance use disorder were reported for 38% of the total prisoner population in Canada (31), 29% in Australia (29), and 21% in Italy (32), lower than in this study.

Forensic populations with the triad of mental disorders, SUD, and antisocial personality disorder may be responsible for more serious and frequent offending (33). Our study showed that people with diagnostic triads in prisons have histories of repeat institutionalizations in psychiatric care and penal justice systems. In South American societies, the removal of psychiatric beds was associated with an increase of prison population rates (34). The crisis in acute psychiatric care may have caused a shift of responsibility to care for people with complex diagnostic triads from psychiatric settings to criminal justice systems. Repeat imprisonments and psychiatric hospitalization in the past may indicate increased risk for future episodes of institutionalization.

The prevalence of a comorbid triad between internalizing disorders (mood and anxiety disorders), substance use disorders, and behavioral disorders (conduct and oppositional defiant disorder if ≤17 years old and antisocial personality disorder if ≥ 18 years old) had been reported for juvenile offenders (14). The triad was present in 11% of the adolescent men and 14% of the adolescent women in juvenile detention. The three diagnostic groups juxtaposed in our study usually require different treatment approaches: SMIs often need repeat psychiatric evaluations and medication (18). PDs may respond to dialectic behavioral therapies and often require long term psychotherapy (35). SUDs are usually treated with relapse prevention, motivational and contingency therapies, medication, and often social and occupational care (36). Active substance use and emotional dysregulation can interfere with any of the treatments. All of the conditions need informant histories and family involvement. Transdiagnostic treatments focusing on core difficulties such as emotional regulation have been proposed (37), and new communication technologies may improve the access to those treatments (38). How to successfully integrate the treatment needs for people with complex comorbidity within the prison context is unresolved. However, imprisonment constitutes an opportunity to offer treatments during a period in life with limited or reduced access to substances to populations that are difficult to reach with outpatient treatment offers in the community. This window of opportunity is currently underused in most systems. Clearly, it is not sufficient to transfer evidence from clinical settings to this highly comorbid population. New intervention trials are needed to test efficacy in this population and setting.

### Strengths and Limitations

To our knowledge this is the first study describing the prevalence of a triad between different clinically relevant groups of disorders in prison populations of Latin America. It was sufficiently powered to assess the effect of a range of sociodemographic and criminal risk factors as well as histories of previous institutionalizations on the outcome. This study has also several limitations. The interview schedule of our study did not include the assessment of neurodevelopmental disorders such as attention deficit/hyperactivity disorder, autism spectrum disorders, and intellectual disability, which may relate to other mental disorders and disruptive behaviors in prison populations (39). It was conducted in Chile, and the findings may not be applicable to other high-income economies. This limitation may apply particularly to women since the prevalence of SUD was considerably lower than in most Western high-income countries (4).

### Conclusions for Research, Practice, and Policy

The complexity and difficulty to treat comorbidity of SMI, SUD, and PD are present in about one out of three people upon imprisonment in Chile. It is especially frequent in young men and individuals with lower educational levels. Routine screening for mental disorders should include those three disorder groups to identify individuals with complex treatment needs and to connect them with psychiatric services during and after imprisonment. Treatment trials specifically targeting people with those complex disorder triads are needed inside prison contexts as well as in psychiatric and community settings. Continuous care models need to be developed involving interdisciplinary and interinstitutional collaborations to break the cycle of repeat institutionalizations in prisons and in hospitals.

## Data Availability Statement

The raw data supporting the conclusions of this article will be made available by the authors, without undue reservation.

## Ethics Statement

The study involving human participants reviewed and approved by ethics committee of the Hospital Clínico Universidad de Chile (Acta de Aprobación 01 from 25.01.2012) and the Ministry of Justice of the Republic of Chile (reference: Subsecretaria de Justicia 15.03.2012). The participants provided their written informed consent to participate in this study.

## Author Contributions

AM conceived of the study. GB conducted the data analyses. AM drafted the manuscript. All authors contributed to the article and approved the submitted version.

## Funding

AM receives funding from the Agencia Nacional de Investigación y Desarrollo ANID (grant scheme: FONDECYT Regular; number 1190613); GB receives funding from the European Union’s Horizon 2020 research and innovation programme under the Marie Skłodowska-Curie grant (agreement number 676060 [LONGPOP]). This publication reflects only the author’s view, and the Research Executive Agency is not responsible for any use that may be made of the information it contains.

## Conflict of Interest

The authors declare that the research was conducted in the absence of any commercial or financial relationships that could be construed as a potential conflict of interest.
